# Impact of Microbial Interactions on Growth and Flavor Profiles of Indigenous Yeasts and Lactic Acid Bacteria in Shanxi Aged Vinegar: Combinatorial Co-Cultures Versus Monocultures

**DOI:** 10.3390/foods15132371

**Published:** 2026-07-03

**Authors:** Xin Zhao, Yujing Zhang, Nv Xu

**Affiliations:** 1 Department of Materials and Chemical Engineering, Taiyuan University, Taiyuan 030032, China; zhaoxin@tyu.edu.cn; 2 Shanxi Province Vinegar Brewing Technology Innovation Center, College of Food Science and Engineering, Shanxi Agricultural University, Jinzhong 030801, China

**Keywords:** Shanxi aged vinegar, yeast, lactic acid bacteria, interaction

## Abstract

Yeasts and lactic acid bacteria, as the predominant microorganisms in Shanxi aged vinegar, play critical roles in shaping its characteristic flavor profile and sensory properties. In this study, we investigated the cooperative fermentation interactions between three yeast strains and two lactic acid bacteria strains isolated from the Shanxi aged vinegar fermentation process. The results showed that *Lactiplantibacillus plantarum* CPL56 and *Pediococcus acidilactici* CPL23 promoted the growth of *Saccharomyces cerevisiae* JLY421 and *Candida artemisiae* JLY1608, while exerting an inhibitory effect on *Saccharomyces cerevisiae* JLQ39. Strains JLY421 and JLY1608 also promoted the growth of CPL56 and CPL23, while JLQ39 and CPL23 enhanced the growth of CPL56. Co-cultivation of strains JLY421 and CPL56 at a 1:1 ratio (*v*/*v*) resulted in significantly higher total acid and organic acid contents compared to all other combinations. Total organic acid content increased by 200.83% and 81.29% relative to the pure cultures of JLY421 and CPL56, respectively, with lactic acid and acetic acid concentrations reaching 0.3278 g/100 g and 0.0970 g/100 g, respectively. Additionally, the total ester content in this co-culture was also significantly higher than in other combinations, representing a 24.48% increase compared to the JLY421 monoculture. Co-cultivation of JLY421 and CPL56 markedly enhanced both the diversity and content of key volatile organic compounds, including isoamyl acetate, isoamyl alcohol, isobutyl acetate, ethanol, amyl butyrate, p-xylene, acetic acid, 3-hydroxy-2-butanone, and 2,3,5,6-tetramethylpyrazine, relative to their respective monocultures. This study provides foundational insight into yeasts and lactic acid bacteria composite starters in a solid sorghum medium, and may offer a useful reference for future efforts to improve the quality and efficiency of Shanxi aged vinegar production in more complete, multi-stage industrial processes.

## 1. Introduction

Vinegar is among the most widely consumed acidic condiments worldwide. According to the raw materials used, vinegars are generally classified into cereal vinegars, derived from starch-rich grains such as sorghum, rice, and wheat, and fruit vinegars, produced from fermentable fruits such as grapes and apples [1]. In China, most vinegars are produced using cereals as the primary raw materials. Shanxi aged vinegar is a representative traditional Chinese cereal vinegar with a history of over 3000 years. It is the only vinegar recognized as a geographically indicated product in both China and Europe [2]. The flavor of Shanxi aged vinegar differs from that of other Chinese cereal vinegars and is characterized by its mellow, sour, soft, rounded, and full-bodied taste. The distinctive flavor of Shanxi aged vinegar is closely associated with its production process, which involves four major stages: alcoholic fermentation, acetic acid fermentation, fumigation, and aging. The entire brewing process occurs through open and spontaneous solid-state fermentation [3,4]. During this process, various microorganisms are naturally inoculated, without the addition of any selected strains.

The microorganisms involved in fermentation—yeasts, acetic acid bacteria, *Bacillus*, lactic acid bacteria, and molds—create a complex and distinctive fermentation system that together enhances the production of bioactive compounds, including organic acids and phenolics, as well as characteristic flavor compounds [5,6]. Among these microorganisms, yeasts are considered critical for the succession of Shanxi aged vinegar fermentation, whereas lactic acid bacteria play a key role in enhancing vinegar flavor [7].

Yeasts dominate the alcoholic fermentation phase of Shanxi aged vinegar and serve as multifunctional agents. During alcoholic fermentation, yeasts produce various secondary metabolites, including organic acids and esters, which play a crucial role in flavor formation [8,9]. Lactic acid bacteria significantly contribute to vinegar quality by not only producing organic acids (e.g., lactic and malic acids) and various amino acids that confer a unique flavor, but also generating flavor-related compounds, specifically 3-hydroxy-2-butanone and 2,3-butanediol, as well as functional metabolites including exopolysaccharides and γ-aminobutyric acid [10,11]. Recent molecular sensory studies on Shanxi aged vinegar have identified specific aroma-active compounds with high odor activity values and have shown that only a subset of the many detected volatiles makes major contributions to the characteristic aroma profile [2,3]. These studies also highlighted that the formation of key aroma-active compounds is closely linked to the succession of microbial communities during alcoholic and acetic acid fermentation, fumigation, and aging [12]. Previous studies have isolated high-performance strains from vinegar fermentation, demonstrating that fortified fermentation with selected indigenous strains alters the vinegar flavor profile. For instance, inoculation with selected yeasts such as *Pichia manshurica* or *Wickerhamomyces anomalus* significantly enriches the volatile profile by elevating ester concentrations and introducing novel aroma compounds [13,14]. Similarly, inoculation with selected lactic acid bacteria such as *Lactobacillus pentosus* during vinegar fermentation optimizes the organic acid composition and elevates levels of bioactive compounds, such as ligustrazine and amino acids [15].

However, vinegar fermentation represents a complex ecosystem in which the metabolic performance of these isolates is fundamentally governed by microbial interactions [6]. The metabolic products of specific microbes often serve as essential substrates for others, thereby influencing overall fermentation efficiency [16]. Therefore, in addition to single-strain inoculation, more studies are now investigating multi-strain consortia. Cooperative interactions among co-cultured microorganisms promote the production of diverse natural bioactive compounds, with co-culture systems yielding higher metabolite levels than monocultures [17,18]. To elevate flavor profile and quality of citrus vinegar, Chen et al. achieved positive results by co-culturing *Saccharomyces cerevisiae* and *Lactiplantibacillus plantarum* during alcoholic fermentation [19]. In another study, Li et al. enhanced volatile ester and total organic acid production in fermented apple-cider vinegar using combined cultures of *Saccharomyces cerevisiae* and *Lactiplantibacillus plantarum* [20]. Moreover, Furukawa et al. found that co-cultivation of *Lactiplantibacillus plantarum* and *Saccharomyces cerevisiae* promotes mutual growth, thereby enhancing metabolic efficiency and flavor development in traditional Fukuyama pot vinegar fermentation [21]. Although numerous studies have investigated co-culture systems of selected yeasts and lactic acid bacteria to improve vinegar fermentation characteristics, they have focused exclusively on liquid-state fermentation, with no relevant research on their synergistic effects in solid-state vinegar fermentation. Moreover, prior co-culture experiments have predominantly employed single strains of each microorganism type, with few studies exploring microbial interactions in multi-strain co-cultures. Against this background, there is still limited information on how indigenous yeasts and lactic acid bacteria, assembled into combinatorial co-cultures, influence both microbial growth and the formation of volatile compounds with potential aroma activity in solid-state Shanxi aged vinegar fermentation.

Previously, we isolated the yeast strains JLY421, JLQ39, and JLY1608, as well as the lactic acid bacteria strains CPL56 and CPL23, from Shanxi aged vinegar fermentation, and these strains showed good fermentation performance in vinegar brewing. In this study, we focused on the interactions between yeasts and lactic acid bacteria in a simulated solid sorghum medium and examined how different strain combinations affected microbial growth and flavor-related metabolite formation. These results provide a useful reference for selecting yeasts and lactic acid bacteria composite starters and offer a scientific basis for future quality improvement of Shanxi aged vinegar in more complete, multi-stage fermentation processes.

## 2. Materials and Methods

### 2.1. Strains

*Saccharomyces cerevisiae* JLY421, *Saccharomyces cerevisiae* JLQ39, *Candida artemisiae* JLY1608, *Lactiplantibacillus plantarum* CPL56, and *Pediococcus acidilactici* CPL23 were obtained from the Shanxi aged vinegar fermentation residue and maintained at the Laboratory of Biological Engineering, College of Food Science and Engineering, Shanxi Agricultural University.

### 2.2. Interaction Between Yeasts and Lactic Acid Bacteria

#### 2.2.1. Preparation of Sorghum Solid-State Medium

The sorghum solid-state medium was prepared by crushing sorghum kernels into 6–8 fragments and mixing them with water to adjust the initial moisture content to approximately 58% (*w*/*w*). The mixture was then conditioned at room temperature for 12 h to ensure full hydration, and the substrate was maintained at its natural pH during this step. The hydrated sorghum was then steamed for 2.0 h, followed by a 20 min holding period, to obtain a fully gelatinized, non-sticky substrate free of raw centers. After that, 50 g of the hot sorghum was transferred to 250 mL Erlenmeyer flasks and mixed with approximately 112.5 mL hot water at 75 °C, keeping the medium at its natural pH. The flasks were then sterilized at 121 °C for 20 min [22].

#### 2.2.2. Interactions of Different Strain Combinations of Yeasts and Lactic Acid Bacteria

The activated lactic acid bacteria strains CPL56 and CPL23 were inoculated into MRS medium (incubated at 37 °C for 2 days), while the yeast strains JLY421, JLY1608, and JLQ39 were cultured in potato dextrose broth (PDB) at 30 °C for 2 days. Lactic acid bacteria: Incubated at 37 °C for 2 days to reach 1 × 10^8^ CFU/mL. Yeasts: Incubated at 30 °C for 2 days to reach 1 × 10^6^ CFU/mL.

Seed cultures of three yeast strains and two lactic acid bacteria strains were combined at defined volume ratios. In total, 16 defined co-culture systems were established. Two-strain combinations comprised six co-cultures—JLY421+CPL56, JLY421+CPL23, JLY1608+CPL56, JLY1608+CPL23, JLQ39+CPL56, and JLQ39+CPL23—each prepared by mixing equal volumes (1:1, *v*/*v*) of the corresponding activated lactic acid bacteria and yeast cultures, followed by co-inoculation at a total inoculum level of 2% (*v*/*w*) into the sorghum solid-state medium. Three-strain combinations included six co-cultures—JLY421+JLY1608+CPL56, JLY421+JLY1608+CPL23, JLY421+JLQ39+CPL56, JLY421+JLQ39+CPL23, JLY1608+JLQ39+CPL56, and JLY1608+JLQ39+CPL23—each prepared by mixing equal volumes of the three starter suspensions (1:1:1, *v*/*v*/*v*) so that each strain contributed one-third of the 2% (*v*/*w*) total inoculum. Four-strain combinations consisted of three co-cultures—JLY421+JLY1608+CPL56+CPL23, JLY421+JLQ39+CPL56+CPL23, and JLY1608+JLQ39+CPL56+CPL23—each prepared at a 1:1:1:1 volumetric ratio, with each strain contributing one-fourth of the 2% (*v*/*w*) total inoculum. The five-strain combination (JLY421+JLY1608+JLQ39+CPL56+CPL23) was prepared at a 1:1:1:1:1 volumetric ratio, with each strain contributing one-fifth of the 2% total inoculum. Each strain was inoculated individually as a control, and each experimental group was conducted with three independent replicates. All solid-state fermentations were carried out at 37 °C for 6 days, after which the fermentation was terminated and samples were collected for analysis.

#### 2.2.3. Biomass Enumeration Techniques

On days 2, 4, and 6, roughly 25 g samples were drawn from the solid-state fermented sorghum. Each sample was suspended in 225 mL of sterile saline with glass beads and homogenized at 140× *g* for 30 min at ambient temperature. Serial dilutions were performed, and 100 μL aliquots of suspensions containing CPL56 or CPL23 were plated on MRS agar and incubated at 37 °C for 48 h. In parallel, 100 μL aliquots of suspensions containing JLY421, JLY1608, or JLQ39 were inoculated onto PDA plates and incubated at 30 °C for 48 h. Plates yielding 30–300 colonies were enumerated, and yeast versus lactic acid bacteria were distinguished based on colony morphology [23].

#### 2.2.4. Determination of Reducing Sugar, Total Acid, Alcohol, and Total Ester

Reducing sugar was quantified by Fehling’s titration, following the Chinese National Standard GB/T 19777-2013 [24]; the total acid content was assessed by titration with 1 mol/L NaOH, according to the Chinese Standard GB/T 19777-2013 [24]; the alcohol content was determined using the alcoholmeter method according to the Chinese National Standard GB 5009.225-2023 [25]; quantification of total ester followed the NaOH saponification method in accordance with the Chinese National Standard GB/T 19777-2013 [24].

#### 2.2.5. Determination of Organic Acids

On day 6, 5 g of the sorghum solid-state fermentation sample was mixed with 15 mL of distilled water for 3 h, and the mixture was then diluted to a final volume of 50 mL with distilled water. After centrifugation (12,000× *g*, 5 min), the supernatant was clarified using a 0.22 µm filter. The concentrations of organic acids (succinic acid, acetic acid, pyruvic acid, oxalic acid, lactic acid, malic acid, tartaric acid, and citric acid) were then determined by the external standard method. High-performance liquid chromatography (HPLC) was performed on a Thermo Fisher UltiMate 3000 system equipped with a 5 µm C18 column (4.6 × 150 mm). The isocratic elution was carried out using 20 mmol/L NaH_2_PO_4_ (adjusted to pH 2.7) as the mobile phase at 0.8 mL/min. A sample volume of 20 µL was injected, and ultraviolet detection was conducted at 210 nm, while the chromatographic column was maintained at laboratory room temperature [26].

#### 2.2.6. Determination of Volatile Aroma Composition and Content

Approximately 5 g of the sorghum solid-state fermentation sample collected on day 6 was placed in a headspace vial, and 1.0 g of NaCl and 10 µL of 0.8775 mg/mL methyl octanoate solution were added. The sample was then subjected to headspace solid-phase microextraction using a 50 µm fiber at 40 °C for 15 min with continuous agitation. The volatile aromatic compounds were analyzed by gas chromatography–mass spectrometry (GC–MS; Agilent 6890N-5973 MSD, Santa Clara, CA, USA) equipped with a VF-5MS capillary column (30 m × 0.25 mm × 0.25 µm; Thermo Fisher Scientific (Waltham, MA, USA). The oven temperature program was as follows: initial temperature 40 °C held for 3 min, then increased at 4 °C/min to 160 °C and held for 1 min, followed by an increase at 10 °C/min to 270 °C and held for 1 min. High-purity helium (99.999%) was used as the carrier gas at a constant flow rate of 1.0 mL/min in splitless mode [27]. The types and semi-quantitative levels of volatile aroma compounds were measured using a direct internal standard approach. Methyl octanoate (0.8775 mg/mL) served as the internal standard, and the relative contents of volatile compounds were calculated based on the ratio of analyte peak area to internal standard peak area. Compound identification was achieved by comparing the acquired mass spectra with the NIST 17.0 mass spectral library, with compounds accepted at a similarity of ≥80%. The GC–MS data are interpreted on a semi-quantitative basis and used to compare relative levels among strain combinations rather than as fully validated absolute concentrations.

### 2.3. Data Analysis

All analyses were conducted in triplicate, with three independent fermentation replicates for each treatment, and the results are presented as mean ± standard deviation (SD). Statistical analyses were performed using SPSS software (version 23.0; IBM Corp., Chicago, IL, USA). For biomass data in pure culture and co-culture systems, two-way ANOVA with strain combination and fermentation time as fixed factors was used, followed by Tukey’s post hoc test. For variables measured at a single time point, one-way ANOVA followed by Tukey’s test was applied to compare differences among strain combinations. In all analyses, statistical significance was defined at *p* < 0.05.

## 3. Results and Discussion

### 3.1. Colony and Cell Morphology

**Figure 1:**
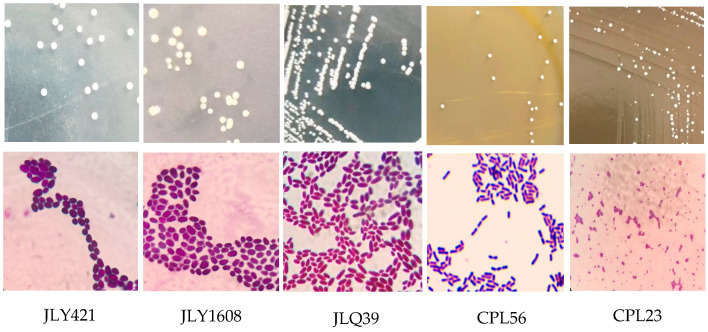
Colony and cell morphology of yeast and lactic acid bacteria strains. Notes: JLY421: *Saccharomyces cerevisiae* JLY421; JLY1608: *Candida artemisiae* JLY1608; JLQ39: *Saccharomyces cerevisiae* JLQ39; CPL56: *Lactiplantibacillus plantarum* CPL56; CPL23: *Pediococcus acidilactici* CPL23.

Representative images of the three yeast strains and two lactic acid bacteria strains are shown in Figure 1, and their colony and cellular morphologies are summarized in Table 1.

**Table 1:** The colony and cell morphology of yeast and lactic acid bacteria strains in Shanxi aged vinegar.

Strains	Cell Morphology	Colonial Morphology
*Saccharomyces cerevisiae* JLY421	Ellipsoidal shape	Colony diameter 2.0 mm, porcelain-white, opaque, glossy surface, a matte texture and lacking distinct moist, sticky appearance, smooth, intact margins, convex elevation.
*Saccharomyces cerevisiae* JLQ39	Ellipsoidal shape	Colony diameter 1.8 mm, milky-white, opaque, moist, sticky surface, creamy luster, regular and circular margins, full central elevation, slightly thinner edge.
*Candida artemisiae* JLY1608	Ellipsoidal shape	Colony diameter 1.0 mm, cream-colored, opaque, moist surface, greasy sheen, soft texture, irregular margins in some colonies, relatively flat elevation.
*Lactiplantibacillus plantarum* CPL56	Slender, short rod	Colony diameter 0.8 mm, regular circles, milky white, opaque, smooth and moist surface, tidy margins, slightly convex, lacking a distinct creamy luster.
*Pediococcus acidilactici* CPL23	Spherical shape	Colony diameter 0.5 mm, bright white, opaque circular, smooth and moist surface, tidy margins, subtly convex dome-shaped elevation.

### 3.2. Biomass in Pure Culture and Co-Culture Systems

**Figure 2:**
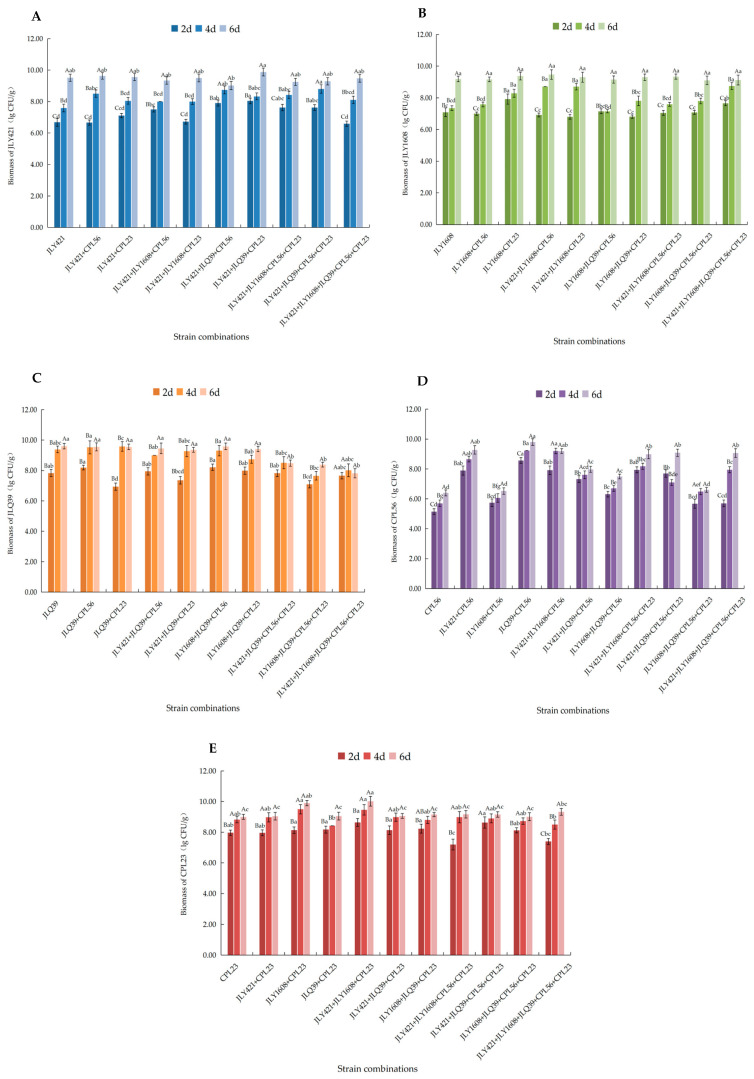
Biomass of yeast and *Lactic acid* bacteria strains in pure culture and co-culture systems. Notes: (**A**) Biomass of *Saccharomyces cerevisiae* JLY421. (**B**) Biomass of *Candida artemisiae* JLY1608. (**C**) Biomass of *Saccharomyces cerevisiae* JLQ39. (**D**) Biomass of *Lactiplantibacillus plantarum* CPL56. (**E**) *Pediococcus acidilactici* CPL23. Data are expressed as mean ± standard deviation (SD). Different letters denote significant differences between groups (*p* < 0.05), whereas identical letters indicate no significant differences (*p* > 0.05). Capital letters indicate changes at different time points within the same strain combination; lowercase letters indicate changes among different strain combinations at the same time point.

The biomasses of strains JLY421, JLY1608, JLQ39, CPL56, and CPL23 were quantified on days 2, 4, and 6 in pure and co-culture systems to investigate growth dynamics. As shown in Figure 2A, during co-cultivation of yeasts and lactic acid bacteria, the biomass of strain JLY421 in the JLY421+JLQ39+CPL56, JLY421+JLQ39+CPL23, and JLY421+JLQ39+CPL56+CPL23 combinations was significantly higher than in pure culture on day 2, increasing by 1.23 lg CFU/g, 1.36 lg CFU/g, and 0.94 lg CFU/g, respectively. In the JLY421+JLY1608+CPL56, JLY421+JLY1608+CPL23, and JLY421+JLY1608+JLQ39+CPL56+CPL23 co-cultures, the biomass of strain JLY1608 on day 4 was significantly higher than in pure culture, increasing by 1.36 lg CFU/g, 1.35 lg CFU/g, and 1.39 lg CFU/g, respectively (Figure 2B). These results indicate that strains CPL56 and CPL23 promoted the growth of JLY421 and JLY1608. This promotion may be related to metabolic cross-feeding, with lactic acid bacteria-derived metabolites (e.g., monosaccharides and amino acids) providing essential carbon and nitrogen sources for yeast [28]. In addition, pH reduction due to organic acid production by lactic acid bacteria has been reported to create a more favorable environment for yeast proliferation, which could also contribute to the observed effects [29]. However, no significant growth promotion was observed for JLQ39 (Figure 2C). This phenomenon may instead reflect nutritional competition between lactic acid bacteria and yeast, which can negatively impact yeast growth. In co-culture systems involving lactic acid bacteria and multiple yeasts, growth promotion or inhibition of specific *S. cerevisiae* strains is likely influenced by their inherent physiological characteristics. Strains with stronger stress tolerance and more flexible metabolic regulation may be better able to utilize metabolites derived from lactic acid bacteria, such as amino acids and vitamins, to support growth, whereas strains with lower acid tolerance and weaker stress resistance are more susceptible to nutritional competition and organic acid accumulation (e.g., lactic acid), leading to growth inhibition [30]. It should be noted that these mechanistic explanations were not directly tested in the present study and should be regarded as plausible interpretations consistent with previous reports, rather than experimentally demonstrated mechanisms.

The biomass of strain CPL56 was consistently higher in all co-culture systems compared to the pure culture (Figure 2D). The biomass of strain CPL56 was consistently higher in all co-culture systems compared with the pure culture (Figure 2D). Specifically, the combinations JLY421+CPL56, JLQ39+CPL56, JLY421+JLY1608+CPL56, JLY421+JLQ39+CPL56, JLY421+JLY1608+CPL56+CPL23, JLY421+JLQ39+CPL56+CPL23, and JLY421+JLY1608+JLQ39+CPL56+CPL23 exhibited increases of 2.88 lg CFU/g, 3.40 lg CFU/g, 2.80 lg CFU/g, 1.57 lg CFU/g, 2.58 lg CFU/g, 2.68 lg CFU/g, and 2.66 lg CFU/g, respectively, on day 6. In the JLY1608+CPL23 and JLY421+JLY1608+CPL23 combinations, the biomass of strain CPL23 was significantly higher than those in the pure culture on both the fourth and sixth days (Figure 2E). Specifically, on the fourth day, the biomass increased by 0.68 lg CFU/g and 0.64 lg CFU/g, respectively. These increases reached 0.90 lg CFU/g and 1.02 lg CFU/g on the sixth day, respectively. These findings indicate that strains JLY421, JLQ39, and JLY1608 promoted the growth of strains CPL56 and CPL23. Furthermore, CPL23 also promoted CPL56 growth. Most lactic acid bacteria are fastidious microorganisms, lacking complete biosynthetic pathways for essential amino acids, vitamins, and other growth factors. Yeasts can potentially compensate for these deficiencies by secreting metabolites or through autolysis, thereby creating a more favorable microenvironment for lactic acid bacteria proliferation. Ponomarova et al. examined the growth dynamics of *S. cerevisiae*, *L. lactis*, and *L. plantarum* in pure and co-culture systems [31]. They found that in CDM35 medium with eight specific amino acids, *S. cerevisiae* biomass showed negligible differences between pure and co-culture systems, whereas *L. lactis* and *L. plantarum* grew only when co-cultured with *S. cerevisiae*. This growth was attributed to yeast secretion of amino acids such as glutamine and serine, which served as essential growth factors for lactic acid bacteria [32]. Mendes et al. reported symbiotic interactions in the *S. cerevisiae*–*Lactobacillus delbrueckii* subsp. *bulgaricus*. co-culture system, in which the lactic acid bacteria hydrolyzed lactose to glucose and galactose to provide available carbon sources for yeast, while yeast metabolism produced CO_2_ to create localized anaerobic conditions favorable for bacterial growth and supplied alanine, thereby enhancing bacterial biomass [33]. These findings are consistent with the hypothesis that the cross-feeding of amino acids between yeasts and the lactic acid bacteria cross-feeding of amino acids, vitamins, and carbon sources may underlie the growth-promoting effects observed in our co-culture systems, although these mechanisms were not directly measured in the present study.

### 3.3. Reducing Sugar Utilization and Production Characteristics of Total Acid, Ethanol, and Total Ester

**Figure 3:**
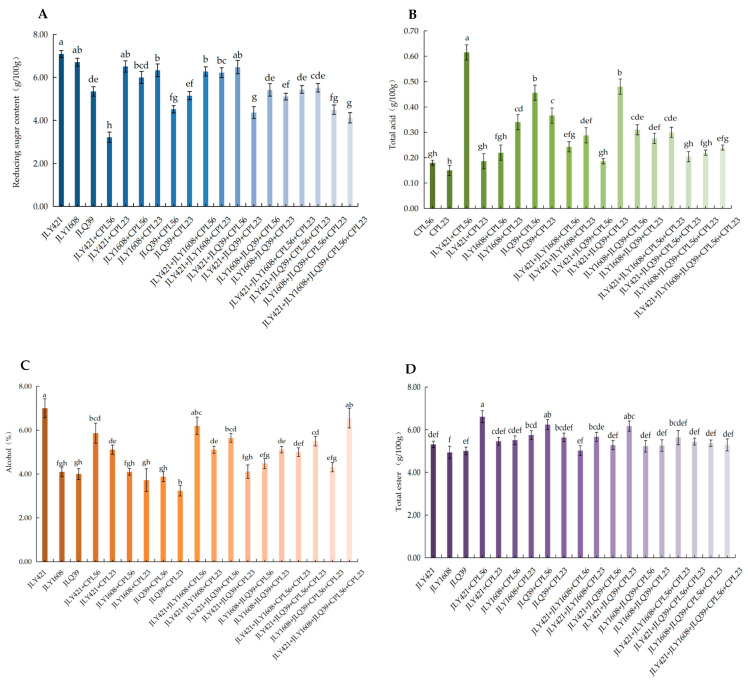
Reducing sugar content, acid production, alcohol production, and ester production in pure culture and co-culture systems. Notes: (**A**) Reducing sugar content. (**B**) Total Acid content. (**C**) Alcohol content. (**D**) Total ester content. Data are expressed as mean ± standard deviation (SD). Different lowercase letters denote significant differences between groups (*p* < 0.05), whereas identical lowercase letters indicate no significant differences (*p* > 0.05).

The reducing sugar, total acid, ethanol, and total ester of strains JLY421, JLY1608, JLQ39, CPL56, and CPL23 were analyzed in both pure and co-culture conditions. As shown in Figure 3A, when yeast strains JLY421, JLY1608, and JLQ39 were co-cultured individually with lactic acid bacteria strains CPL56 and CPL23, reducing sugar levels decreased overall. The JLY421+CPL56 combination showed the lowest reducing sugar content (3.22 ± 0.24 g/100 g), which was significantly different (*p* < 0.05) and 54.58% lower than that of the JLY421 monoculture. These results indicate that co-culturing yeast with lactic acid bacteria promotes sugar metabolism [34]. This was followed by the JLQ39+CPL56, JLY421+JLQ39+CPL23, JLY1608+JLQ39+CPL56+CPL23 and JLY421+JLY1608+JLQ39+CPL56+CPL23 systems, with reducing sugar contents of 4.52 g/100 g, 4.37 g/100 g, 4.50 g/100 g, and 4.12 g/100 g, respectively. In pairwise co-cultures, combinations containing CPL56 (JLY421+CPL56, JLY1608+CPL56, and JLQ39+CPL56) showed lower reducing sugar levels than their corresponding combinations with CPL23 (JLY421+CPL23, JLY1608+CPL23, and JLQ39+CPL23), with reductions of 50.54%, 5.21%, and 12.23%, respectively. This suggests that CPL56 exhibits stronger synergistic interactions with yeast than CPL23, leading to greater carbon source consumption. As the proportion of yeast increased, ethanol accumulated through alcoholic fermentation. This ethanol disrupted the cell membrane integrity and sugar transport systems of lactic acid bacteria, thereby inhibiting reducing sugar consumption. Therefore, the yeast proportion in co-culture is a key factor influencing sugar metabolism in the system.

In Shanxi aged vinegar fermentation, total acid content is a critical quality parameter that determines sensory profile and flavor while reflecting organic acid composition and fermentation progress [35]. As shown in Figure 3B, total acid content in all co-cultures was consistently higher than in corresponding pure cultures. Notably, JLY421+CPL56 achieved the highest total acid content (0.62 ± 0.03 g/100 g), increasing by approximately 244.44% than CPL56 pure culture. Similarly, JLQ39+CPL56 and JLY421+JLQ39+CPL23 showed substantial increases (155.56% and 220%, respectively) compared to CPL56 and CPL23 pure cultures. Consistent with the enhanced biomass of CPL56 and CPL23 in co-cultures, higher viable cell counts directly promoted total acid accumulation. In these combinations, the reducing sugar contents were also low, with JLY421+CPL56 showing the lowest residual sugar, which is in line with the sugar-to-acid conversion relationship whereby greater sugar consumption corresponds to higher acid production. The superior performance of JLY421+CPL56 suggests that this relatively simple 1:1 consortium forms an efficient and complementary metabolic partnership, in which JLY421 provides strong fermentative activity and precursors, while CPL56 channels carbon into organic acid synthesis without excessive competition from additional strains. By contrast, when four or five strains were co-cultured, increased competition for fermentable substrates among yeasts and lactic acid bacteria, together with the accumulation of metabolic byproducts, may have suppressed the activity of key acid-producing strains through nutrient depletion, antagonistic interactions, or feedback inhibition, resulting in no further improvement in total acid production.

As shown in Figure 3C, ethanol contents in pure cultures of JLY421, JLY1608, and JLQ39 were 7.0 ± 0.43%, 4.1 ± 0.22%, and 4.0 ± 0.25% (*v*/*v*), respectively. JLY421 produced the highest ethanol level, with a significant difference (*p* < 0.05), indicating that JLY421 has a stronger ethanol-producing capacity than the other yeasts. Analysis of alcohol production across different strain combinations revealed that the JLY421+CPL56, JLY421+JLY1608+CPL56, and JLY421+JLY1608+JLQ39+CPL56+CPL23 systems exhibited relatively higher alcohol yields, with ethanol contents reaching 5.86 ± 0.46%, 6.20 ± 0.40%, and 6.55 ± 0.45% (*v*/*v*), respectively. However, alcohol production in these co-culture systems remained lower than that observed in the JLY421 monoculture fermentation. In addition to possible esterification reactions, this reduction may be related to lactic acid bacteria competing with yeasts for fermentable sugars and thereby diverting part of the carbon flux from ethanol to organic acid production. The accumulation of lactic acids in co-cultures may also lower the environmental pH and impose mild acid stress on yeasts, which can modulate glycolytic flux and redirect metabolism away from ethanol and higher alcohol formation. Nicomrat et al. reported that co-inoculation of indigenous lactic acid bacteria from banana vinegar fermentation with yeast resulted in lower ethanol content than in yeast pure culture, which is consistent with our findings [36]. Among the co-culture combinations above, JLY421 stands out as a highly fermentative yeast with a leading metabolic role, capable of maintaining relatively stable ethanol production in both pure culture and co-culture systems despite the presence of lactic acid bacteria.

As shown in Figure 3D, total ester content was consistently higher in all co-cultures than in corresponding pure cultures. Notably, JLY421+CPL56 achieved the highest ester content (6.61 ± 0.28 g/100 g), representing a 24.48% increase compared with the JLY421 pure culture. JLQ39+CPL56 followed with a 24.80% increase over JLQ39 pure culture. The co-culture JLY421+JLQ39+CPL23 increased ester production by 16.20% and 23.4% compared with the JLY421 and JLQ39 monocultures, respectively. Overall, CPL56 and CPL23 synergistically enhanced ester production by JLY421, JLQ39, and JLY1608 in co-culture. In monoculture, yeasts produce more ethanol but less acid, while lactic acid bacteria produce more acid and little ethanol. When combined, their complementary metabolic activities increased the total ester content by providing both alcohols from yeasts and organic acid precursors from lactic acid bacteria. In more complex multi-species co-cultures, however, the diversity of metabolic byproducts from multiple strains may suppress specific ester-synthesizing enzymes, and stronger interspecies competition for substrates can partially offset the acid and alcohol supply needed for ester formation. Li et al. showed that in *S. cerevisiae* and *L. plantarum* cider vinegar co-cultures, the lactic acid served as a key ester synthesis precursor that, through esterification with yeast-derived alcohols, significantly enhanced both ester diversity and content compared to yeast pure culture [20]. This is consistent with our observation that co-cultivation of yeasts and lactic acid bacteria promotes the biosynthesis of volatile esters in the present model system.

### 3.4. Characteristics of Organic Acids in Pure Culture and Co-Culture Systems

**Figure 4:**
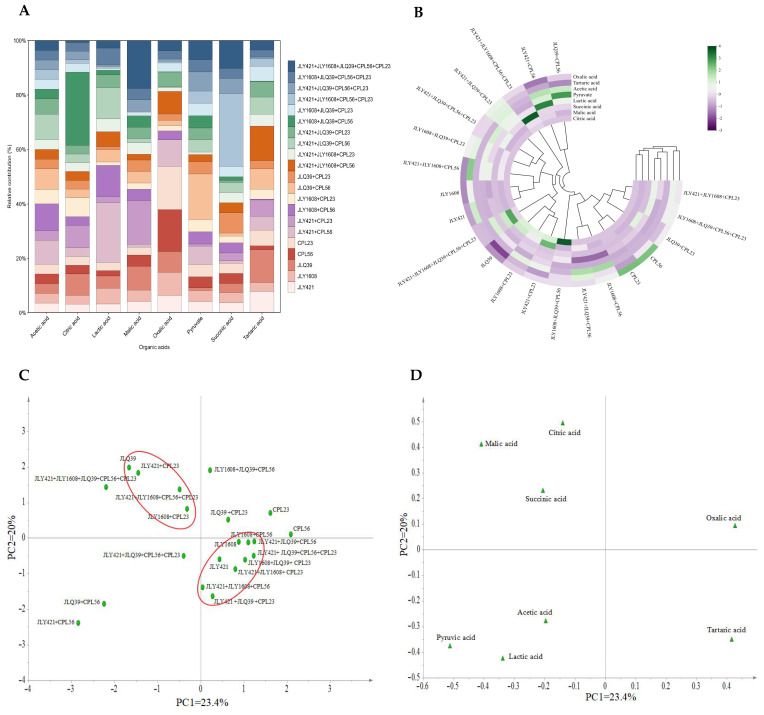
Characterization of organic acid profiles in pure and co-culture systems. Notes: (**A**) Histogram analysis of organic acid content changes. (**B**) Heat map showing the content distribution and hierarchical clustering of organic acids. (**C**) PCA score plot illustrating the differentiation of various culture systems based on their organic acid compositions. The red circle indicates the main cluster of high organic acid‑producing culture systems. (**D**) PCA loading plot identifying the key organic acids contributing to the culture systems.

Organic acids produced during Shanxi aged vinegar fermentation are key taste-active compounds. Together with other flavor compounds, they define the unique flavor profile of Shanxi aged vinegar. These organic acids are primary flavor components that shape the vinegar’s unique sensory profile. Their composition and concentrations directly determine Shanxi aged vinegar quality and sensory characteristics. As shown in Figure 4A,B, organic acid profiles and contents varied significantly across strain combinations. Hierarchical clustering analysis categorized the interactions between yeasts and lactic acid bacteria into two distinct groups. Among all combinations, JLY421+CPL56 showed the highest total organic acid content (0.4690 ± 0.02 g/100 g), which was 200.83% and 81.29% higher than in the JLY421 and CPL56 monocultures, respectively. Within this co-culture, lactic and acetic acids were the most abundant organic acids (0.3278 ± 0.04 and 0.0970 ± 0.011 g/100 g, respectively). Lactic acid, the primary non-volatile acid in fermented vinegar, moderates sourness for smoother taste. Higher non-volatile acid content reduces acidic pungency. In high-quality vinegars, acetic acid is the predominant organic acid, typically accounting for more than 75% of the total acidity, while lactic acid generally constitutes over 15% of the total acidity [37]. Li et al. utilized a co-culture of *S. cerevisiae* and *L. plantarum* for cider vinegar fermentation [20]. Their study showed higher total organic acid content in co-culture than in yeast pure culture. Furthermore, the content of specific organic acids, notably lactic and acetic acids, were substantially elevated. These results align with our findings. Acetic and pyruvic acid contents in JLQ39+CPL56 were significantly higher than in JLQ39 and CPL56 pure cultures. JLY421+CPL23 and JLY421+JLY1608+JLQ39+CPL56+CPL23 showed high levels of malic acid. JLY421+JLY1608+CPL56+CPL23 and JLY421+JLY1608+JLQ39+CPL56+CPL23 showed high levels of succinic acid. Malic acid imparts a smooth, mild sourness with subtle aroma and bitterness, contributing to a lingering aftertaste, while succinic acid combines sourness with umami-like bitterness [38]. Synergistic interactions among these organic acids form vinegar’s unique flavor profile, enhancing overall quality; synergistic interactions among these organic acids collectively shape vinegar’s unique flavor profile and enhance its overall quality.

Figure 4C,D present principal component analysis (PCA) results for organic acid profiles from pure and co-culture systems. The horizontal axis (PC1) accounted for 23.4% of total variance. The vertical axis (PC2) explained 20% of variance. PC1 and PC2 together explained 43.4% of the total variance. Within this portion of the variance, the PCA score plot showed that JLQ39+CPL23, CPL56, CPL23, and JLY1608+JLQ39+CPL56 tended to cluster in the first quadrant, where they were associated with oxalic acid. This pattern is consistent with the relatively high oxalic acid production of CPL56 and CPL23 and may be further modulated by yeast interactions. Cho et al. isolated *Lactobacillus acidophilus*, *L. plantarum*, and *Lactobacillus casei* from veterinary probiotics to evaluate their in vitro oxalate-related properties [39]. Their results indicated that *L. plantarum* significantly increased oxalic acid concentrations, reflecting a strain-specific tendency for oxalate accumulation in vitro, which is in line with the association of CPL56 with oxalic acid observed in our PCA. Li et al. investigated organic acid production by various lactic acid bacteria strains in simulated Shanxi aged vinegar fermentation medium, and reported that *P. acidilactici* L729 produced the highest oxalic acid levels among the tested lactic acid bacteria [38]. Similarly, our results for CPL23 are consistent with their findings. In the PCA score plot, JLQ39, JLY421+CPL23, JLY1608+CPL23, JLY421+JLY1608+CPL56+CPL23, and JLY421+JLY1608+JLQ39+CPL56+CPL23 clustered in the second quadrant, suggesting broadly similar organic acid profiles within the variation captured by PC1 and PC2. The loading plot indicated positive associations with malic, succinic, and citric acids in this region. JLY421+CPL56, JLQ39+CPL56, and JLY421+JLQ39+CPL56+CPL23 were located in the third quadrant, where they were associated with acetic, lactic, and pyruvic acids. In the PCA score plot, JLY1608, JLY421+JLQ39+CPL56, JLY1608+CPL56, JLY421+JLQ39+CPL56+CPL23, JLY421, JLY1608+JLQ39+CPL23, JLY421+JLY1608+CPL23, JLY421+JLY1608+CPL56, and JLY421+JLQ39+CPL23 appeared in the fourth quadrant, which showed a positive association with tartaric acid. This distribution is consistent with the relatively higher tartaric acid levels observed for strains JLY421 and JLY1608 and their combinations.

### 3.5. Characteristics of Volatile Aroma Compounds in Pure Culture and Co-Culture Systems

Volatile aroma compounds are key determinants of vinegar flavor, quality, and sensory acceptance. Their diversity and abundance define vinegar’s characteristic aroma profile and flavor identity. Using CPL56, CPL23, JLY421, JLQ39, and JLY1608 pure cultures as controls, we investigated volatile aroma compound production in their co-cultures. A total of 61 volatile aroma compounds were identified across pure cultures and co-cultures of CPL56, CPL23, JLY421, JLQ39, and JLY1608. These included 17 esters, 3 aldehydes, 13 alcohols, 6 acids, 4 ketones, 5 phenols, 3 pyrazines, and 10 other compounds.

**Figure 5:**
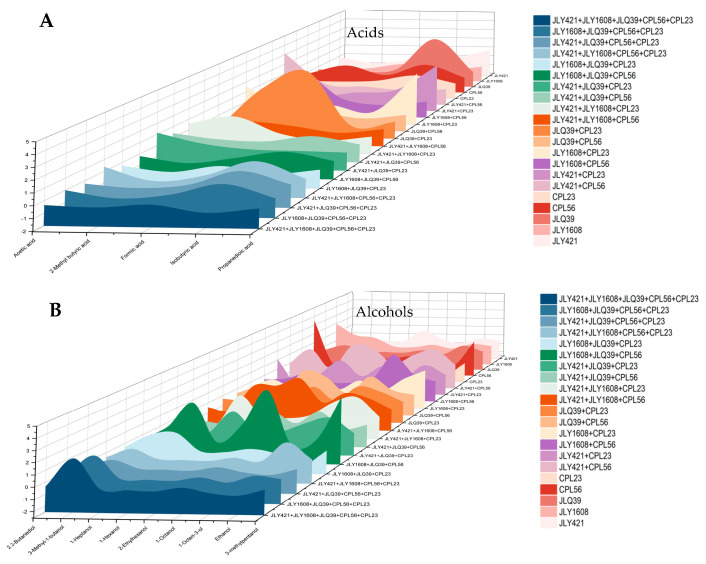

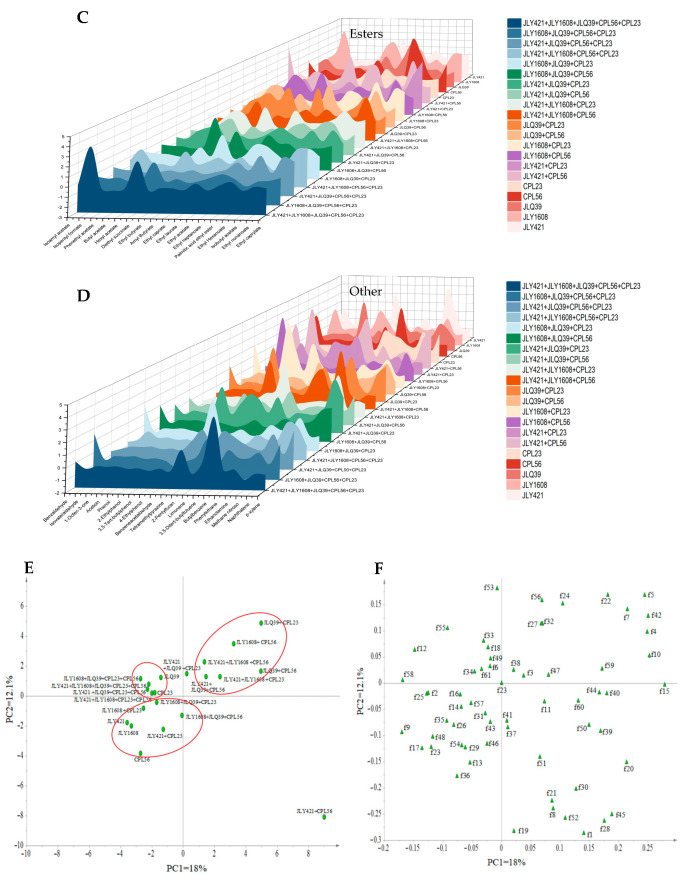
Characterization of volatile aroma compounds in pure and co-culture systems. Notes: (**A**) Comparative analysis of the variety and content of acids. (**B**) Comparative analysis of the variety and content of alcohols. (**C**) Comparative analysis of the variety and content of esters. (**D**) Comparative analysis of the variety and content of other types. (**E**) PCA score plot illustrating the differentiation of various culture systems based on their volatile aroma compounds. The red circle indicates the main cluster of culture systems with high volatile aroma compound production. (**F**) PCA loading plot identifying the key volatile aroma compounds contributing to the culture systems. Abbreviations for volatile aroma compounds: f1: Isoamyl acetate; f2: Isoamyl formate; f3: Phenethyl acetate; f4: Butyl acetate; f5: Hexyl acetate; f6: Diethyl succinate; f7: Ethyl butyrate; f8: Pentyl butyrate; f9: Ethyl decanoate; f10: Ethyl laurate; f11: Ethyl acetate; f12: Ethyl heptanoate; f13: Ethyl palmitate; f14: Ethyl hexanoate; f15: Isobutyl acetate; f16: Ethyl nonanoate; f17: Ethyl octanoate; f18: Benzaldehyde; f19: Isoamaldehyde; f20: Decanal; f21: Acetic acid; f22: 2-Methylbutanoic acid; f23: Isovaleric acid; f24: Formic acid; f25: Isobutyric acid; f26: Malonic acid; f27: 1-Octen-3-one; f28: 3-Hydroxy-2-butanone; f29: 4-Methyl-2-heptanone; f30: Carvone; f31: Phenol; f32: 2-Ethylphenol; f33: 3,5-Di-tert-butylphenol; f34: 3-Ethylphenol; f35: 4-Ethylphenol; f36: 2,3-Butanediol; f37: Isoamyl alcohol; f38: n-Heptanol; f39: n-Hexanol; f40: 2-Ethylhexanol; f41: 1-Octanol; f42: 1-Octen-3-ol; f43: 2-Pentanol; f44: Ethanol; f45: 2-Nitroethanol; f46: 3-Methylpentanol; f47: Furfuryl alcohol; f48: Phenethyl alcohol; f49: 2-Ethyl-3-methylpyrazine; f50: 2,3,5,6-Tetramethylpyrazine; f51: 2,3,5-Trimethylpyrazine; f52: 2-n-Pentylfuran; f53: Limonene; f54: 3,5-Di-tert-butyl-toluene; f55: Butylbenzene; f56: Pentylbenzene; f57: Ethanolamine; f58: Nitrosomethane; f59: Naphthalene; f60: P-xylene; f61: Styrene.

As illustrated in Figure 5A–D, significant differences were observed in both the types and contents of volatile aromatic compounds among the microbial strains. Among the co-culture systems, the JLY421+CPL56 co-culture showed the highest levels of esters, acids, ketones, and alcohols, reaching 1.0438 ± 0.17 mg/100 g, 0.9357 ± 0.11 mg/100 g, 0.1612 ± 0.012 mg/100 g, and 1.1146 ± 0.23 mg/100 g, respectively. In addition, the contents of ester compounds (such as isoamyl acetate, isobutyl acetate, and amyl butyrate), alcohols (such as 1-hexanol, isoamyl alcohol, and ethanol), and other volatiles including p-xylene, acetic acid, 3-hydroxy-2-butanone, 2-pentylfuran, 2,3,5,6-tetramethylpyrazine, and 2,3,5-trimethylpyrazine were significantly higher in the JLY421+CPL56 co-culture than in the JLY421 and L7 monocultures. Isoamyl acetate is typically associated with a characteristic banana-like aroma, whereas isobutyl acetate has been described as contributing ripe-fruit notes in vinegar and other fermented products [40]. Isoamyl acetate has been reported to exhibit a very low odor threshold in the low μg/L range and to possess high OAV values in Shanxi aged vinegar, indicating that even moderate changes in its content can noticeably affect perceived banana-like notes [2]. Acetoin acts as a biosynthetic precursor of tetramethylpyrazine. As a key flavor compound in Shanxi aged vinegar, tetramethylpyrazine has been reported to enhance sensory characteristics by imparting a smooth, mellow acidity with a subtle, rounded sweetness [2]. Tetramethylpyrazine has likewise been identified as a key aroma-active compound with high flavor dilution factors and OAV values above 1, contributing prominently to roasted, nutty, and mellow characteristics in Shanxi aged vinegar [2,3]. The co-cultures JLY1608+CPL56, JLQ39+CPL56, JLY421+JLY1608+CPL23, and JLQ39+CPL23 exhibited elevated contents of multiple volatile aromatic compounds, such as phenethyl acetate, butyl acetate, hexyl acetate, isobutyl acetate, ethyl butyrate, and 2-methylbutyric acid. The JLY421+CPL23 co-culture exhibited increased levels of ethyl nonanoate, ethyl octanoate, and malonic acid, whereas the JLY1608+CPL23 co-culture contained higher concentrations of ethyl hexanoate, 4-ethylphenol, and phenol. The characteristic volatile composition of vinegar is thought to arise from the combined effects of many compounds, which together shape the distinctive aroma profiles of different vinegars. Acids represent an essential core of the volatile aroma profile. Acetic acid, the predominant organic acid, is generally considered the principal contributor to the sour and pungent character of vinegar, while other organic acids such as lactic acid and isovaleric acid can buffer its sharpness and are associated with a milder and more rounded acidity [41].

Esters are often regarded as important contributors to vinegar aroma because many of them have relatively low odor thresholds. Among them, ethyl acetate and isoamyl acetate have been reported to exhibit odor thresholds in the low mg/L and μg/L range, respectively, and are associated with pleasant fruity and banana-like aromas. Furthermore, the accumulation of such esters during aging has been associated with increased perceived richness and depth of vinegar aroma [3]. Alcohols primarily exert a moderating effect on the overall flavor, with ethanol effectively reducing the pungency of acetic acid. Higher alcohols contribute floral notes and serve as essential precursors in ester biosynthesis. Specifically, higher alcohols such as phenethyl alcohol and isoamyl alcohol impart pleasant floral and fruity aromas while simultaneously enhancing aromatic complexity by supplying the necessary substrates for ester formation. Aldehydes and ketones are mainly generated through protein degradation and the Maillard reaction [42]. These compounds have been reported to impart nutty, creamy, and roasted nuances and to contribute to the perceived flavor complexity of aged vinegars. They also contribute substantially to the sensory richness and fullness of aged vinegar. Heterocyclic and phenolic compounds are regarded as characteristic markers of traditional vinegars; for instance, tetramethylpyrazine and 4-ethylguaiacol are linked to smoky and roasted notes that help define the regional identity and uniqueness of specific vinegars [43].

As shown in Figure 5E,F, principal component analysis (PCA) was conducted based on the volatile aromatic compounds identified in both pure and co-culture systems. The horizontal axis represents the first principal component (PC1), explaining 18% of the total variance, while the vertical axis (PC2) accounted for 12.1%. Together, these two principal components explained a cumulative 30.1% of the total variance. In the PCA score plot, the co-cultures JLQ39+CPL23, JLY1608+CPL56, JLY421+JLY1608+CPL56, JLY421+JLQ39+CPL56, JLQ39+CPL56, and JLY421+JLY1608+CPL23 were located in close proximity in the first quadrant, suggesting broadly similar volatile aromatic profiles. These co-cultures showed positive associations with a range of esters, acids, and alcohols, including phenethyl acetate, butyl acetate, hexyl acetate, ethyl butyrate, ethyl laurate, 2-methylbutanoic acid, formic acid, 1-octen-3-one, 2-ethylphenol, n-heptanol, 1-octen-3-ol, furfuryl alcohol, pentylbenzene, and naphthalene. The co-cultures JLQ39, CPL23, JLY1608+JLQ39+CPL23+CPL56, JLY421+JLY1608+CPL23+CPL56, JLY421+JLQ39+CPL23+CPL56, and JLY421+JLY1608+JLQ39+CPL23+CPL56 were grouped in the second quadrant, showing associations with diethyl succinate, ethyl heptanoate, benzaldehyde, 3,5-di-tert-butylphenol, 3-ethylphenol, 2-ethyl-3-methylpyrazine, limonene, butylbenzene, nitrosomethane, and styrene. The strains JLY421, JLY1608, CPL56, JLY1608+CPL23, JLY1608+JLQ39+CPL56, JLY1608+JLQ39+CPL23, and JLY421+CPL23 formed a cluster in the third quadrant, and their associated volatile aromatic profiles were correlated with a variety of compounds, including esters (e.g., isoamyl formate, ethyl hexanoate, ethyl nonanoate), acids (e.g., malonic acid), alcohols (e.g., 4-ethylphenol, 2,3-butanediol, phenethyl alcohol), and phenols (e.g., phenol, 4-ethylphenol). In the PCA score plot, JLY421+CPL56 clustered in the fourth quadrant, correlated with 2,3,5,6-tetramethylpyrazine, n-hexanol, decanal, 2-nitroethanol, and other flavor components such as aldehydes, alcohols, and pyrazine derivatives. The strain CPL56 was characterized by correlations with several representative volatile compounds, including 2,3-butanediol, ethyl octanoate, ethyl decanoate, isovaleric acid, and 3,5-di-tert-butyltoluene. Compared with strain CPL56, the co-culture JLY421+CPL56 generated additional key volatile aromatic compounds, including isoamyl acetate (banana-like), ethyl acetate (fruity), carvone (caraway/dill-like), isoamyl alcohol (apple brandy/spicy), 1-octanol (waxy/citrus), acetic acid (pungent), and 3-hydroxy-2-butanone (buttery/creamy), decanal (waxy orange peel). The presence of these compounds enriched the overall volatile aromatic profile.

The strains CPL56, CPL23, JLY421, JLQ39, and JLY1608 exhibited distinct volatile aroma profiles in both composition and abundance. Compared with pure cultures of individual yeasts or lactic acid bacteria, co-cultures combining yeasts and lactic acid bacteria at varying ratios substantially increased the diversity and abundance of volatile aromatic compounds. In co-culture, ethanol produced by yeasts via alcoholic fermentation provides ample substrates for ester synthesis by lactic acid bacteria. Simultaneously, organic acids produced by lactic acid bacteria modulate the fermentation pH, creating an optimal environment for yeast metabolism. Furthermore, these microbial groups engage in synergistic interactions, including complementary nutrient utilization, mutual metabolite induction, and regulation via quorum sensing signaling molecules [44]. These mechanisms collectively promote the biosynthesis of key aroma compounds, including esters and higher alcohols. Future research should focus on isolating and screening strains with high synergistic potential and superior aroma-producing capacity from traditional aged vinegar fermentation systems. This approach would facilitate the establishment of standardized microbial resource libraries and the optimization of strain combinations. In future work, the integration of multi-omics technologies such as genomics and metabolomics could be used to elucidate the metabolic regulatory mechanisms in co-cultures, thereby deepening our understanding of microbial interactions and supporting more targeted, precise strain engineering.

## 4. Conclusions

This study established pure and co-culture systems using three yeast strains and two lactic acid bacteria strains previously isolated from Shanxi aged vinegar fermentation and cultivated them for 6 days on a simulated solid sorghum medium to investigate interactions among different yeasts and lactic acid bacteria combinations. Results showed that, at the same fermentation time, the biomass of all strains in co-culture systems with different interaction ratios, except *Saccharomyces cerevisiae* JLQ39, was higher than in their respective pure cultures. Further analysis of acid, alcohol, and ester production revealed that co-cultures produced significantly higher levels of acids and esters than pure cultures. In contrast, alcohol production was lower in co-cultures. A total of 61 volatile aroma compounds were identified across all pure and co-culture systems. These comprised 17 esters, 3 aldehydes, 13 alcohols, 6 acids, 4 ketones, 5 phenols, 3 pyrazines, and 10 other compounds. Co-cultures exhibited greater diversity and higher abundance of volatile aroma compounds than pure cultures. Specifically, the 1:1 co-culture of *Saccharomyces cerevisiae* JLY421 and *Lactiplantibacillus plantarum* CPL56 exhibited the highest levels of esters, acids, ketones, and alcohols. Overall, the JLY421+CPL56 co-culture showed the most favorable performance in simulated solid sorghum medium, and the strain combinations identified in this work provide a useful basis for selecting promising candidates for further validation in multi-stage Shanxi aged vinegar fermentation.

## Data Availability

The original contributions presented in the study are included in the article. Further inquiries can be directed to the corresponding author.
